# Methods for medical device and equipment procurement and prioritization within low- and middle-income countries: findings of a systematic literature review

**DOI:** 10.1186/s12992-017-0280-2

**Published:** 2017-08-18

**Authors:** Karin Diaconu, Yen-Fu Chen, Carole Cummins, Gabriela Jimenez Moyao, Semira Manaseki-Holland, Richard Lilford

**Affiliations:** 1 Institute for Applied Health Research, University of Birmingham, B15 2TT, Edgbaston, West Midlands UK; 2 Warwick Centre for Applied Health Research and Delivery, University of Warwick, Coventry, CV4 7AL UK; 3 Institute for Global Health and Development, Queen Margaret University, Edinburgh, EH21 6UU UK; 4 Medicins Sans Frontieres, Artsen Zonder Grenzen, Rue de l’Arbre Benit 46, 1050 Bruxelles, Belgium

**Keywords:** Medical devices, Prioritization, Resource allocation, Equipment, Health technology assessment

## Abstract

**Background:**

Forty to 70 % of medical devices and equipment in low- and middle-income countries are broken, unused or unfit for purpose; this impairs service delivery to patients and results in lost resources. Undiscerning procurement processes are at the heart of this issue.

We conducted a systematic review of the literature to August 2013 with no time or language restrictions to identify what product selection or prioritization methods are recommended or used for medical device and equipment procurement planning within low- and middle-income countries. We explore the factors/evidence-base proposed for consideration within such methods and identify prioritization criteria.

**Results:**

We included 217 documents (corresponding to 250 texts) in the narrative synthesis. Of these 111 featured in the meta-summary. We identify experience and needs-based methods used to reach procurement decisions. Equipment costs (including maintenance) and health needs are the dominant issues considered. Extracted data suggest that procurement officials should prioritize devices with low- and middle-income country appropriate technical specifications – i.e. devices and equipment that can be used given available human resources, infrastructure and maintenance capacity.

**Conclusion:**

Suboptimal device use is directly linked to incomplete costing and inadequate consideration of maintenance services and user training during procurement planning. Accurate estimation of life-cycle costing and careful consideration of device servicing are of crucial importance.

**Electronic supplementary material:**

The online version of this article (doi:10.1186/s12992-017-0280-2) contains supplementary material, which is available to authorized users.

## Background

The absence of safe, effective and well-functioning medical devices and equipment (MDEs) impairs health service provision, leads to poor patient outcomes and poses substantial health system and national security risks [1–3]. Nowhere has this been more evident than the recent Ebola Virus Disease outbreak in West Africa [4–6]. The absence of laboratory equipment to enable quick and accurate diagnosis, and personal protective equipment to ensure effective infection prevention and control measures and health worker safety, directly resulted in delays to emergency response, difficulties in care delivery and lost patient and health worker lives [6].

The absence of appropriate technologies impairs more than emergency care, however: routine services in maternal, child and reproductive care (e.g. immunizations or reproductive control), interventions for non-communicable (e.g. diabetes management) and communicable diseases (e.g. HIV/AIDS diagnosis) all require suitable infrastructure and functioning technologies. From basic products such as weighing scales and condoms, to glucometers and flow-cytometers, health service delivery is predicated upon the availability, appropriateness, affordability and acceptability of MDEs [1].

Estimates suggest that between 40 and 70% of MDEs in resource poor settings are broken, unused or unfit for purpose [7]. Indiscriminate procurement methods, a mismatch in technology design and demand, high costs as well as deployment, maintenance and human resource training challenges all contribute towards this issue [1]. Low- and middle-income countries (LMICs) particularly lack the regulatory authorities, or biomedical engineering capacity, to advise on what MDEs are suitable for use in harsh deployment settings: i.e. facilities with high temperature, fluctuating electricity or no clean water supply. The problem is compounded by a mismatch in MDE supply: manufacturers are located and attuned to users in lucrative high-income markets [8]. Installation, preventive and corrective maintenance services and user training programs are also traditionally absent in LMICs, leading to unsafe device handling practices with potentially harmful consequences for patients (e.g. in cases of misdiagnosis due to mis-calibration or infection propagation due to device re-use).

This paper targets one aspect of the above problem: MDE procurement and resource allocation in LMICs. To assist decision-makers in conducting informed and evidence-based product selection decisions, we have conducted a systematic review of internationally recommended methods for procurement planning and prioritization in settings experiencing severe resource constraints, describing the context for these activities and summarising normative recommendations. Little is known about how MDE procurement takes place within resource-constrained settings; the WHO Baseline Surveys on Medical Devices are recent attempts to explore this [9, 10]. The survey’s findings suggest that LMICs predominantly conduct procurement at central ministry level within the public sector [9]; however, the surveys do not provide further granular information on how LMIC conduct or plan MDE procurement. For the purposes of this paper, we adopt the MDE definition laid out by the Global Harmonization Task Force and WHO [11, 12].

The identification of best practices and common pitfalls in MDE procurement may lead to improvements in MDE management and use. This would not only ensure improved use of scarce financial resources, but also translate to devices being operational and used competently for improved patient care. The systematic review is particularly timely given global efforts for health system strengthening in LMICs: MDEs are recognized as critical components in ensuring health system resilience to shocks and in achieving universal health coverage. Substantial increases in MDE utilization have already been noted in middle-income countries in Europe in line with expanding provision of health care services; similar trends will undoubtedly follow in LMICs globally [13].

We proceed to report the methods and findings of a systematic literature review of the LMIC relevant MDE literature aiming to explore the following research questions:

1. Who are the key stakeholders engaged in procurement planning and what activities/interactions do they engage in?
2. What methods inform procurement planning?
3. What factors are considered in procurement planning?
4. What factors affect successful deployment and use of MDEs?
5. Where specific prioritisation algorithms exist to guide procurement, what criteria do said algorithms use?
6. What are current recommendations for improving procurement?

## Methods

A full account of methods is available in the study protocol [14].

### Searches and study selection

**Fig. 1:**
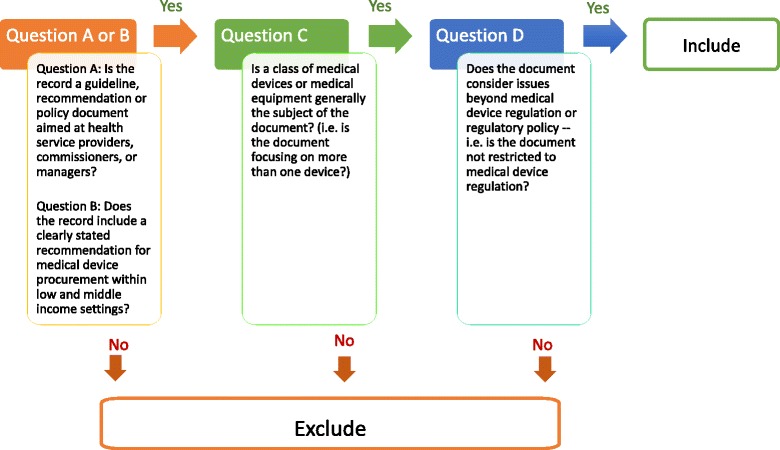
Abstract selection algorithm and criteria*. * All abstracts were reviewed in light of the above questions

We searched both bibliographic databases and grey literature up to August 2013 with no language or time restrictions imposed and selected documents according to pre-specified screening and eligibility criteria. Table 1 lists sources searched and Fig. 1 illustrates abstract selection criteria; Additional files 1 and 2 include details on the OVID MEDLINE search string and search and selection algorithm used.

**Table 1:** Sources searched

Search type	Search sources	
OVID Medline searched as per search algorithm detailed in protocol	Bibliographic databases	OVID Medline, OVID Embase, Cochrane Library, CEA Registry, HMIC, Econlit, VHL Portal (includes LILACS), African Index Medicus, NHS EED, Web of Science (including CPCI)
Key word searches	Website searches	TRIP, National Guideline Clearinghouse, Office of health economics International Guideline Library, CHEPA, CHE York HTAi, CADTH, INAHTA
	Organizational databases/websites	WHO e-health documentation centre and WHO website, UNICEF, UNAIDS UNFPA, African development bank, Asian Development Bank, EBRD, World Bank, MSF, UNDP, UNFPA
	National/regional donor or research agencies	DFID, MSH, AUSAID, GIZ, BMZ, JICA, SWISSAID, CIDA (Canada), DANIDA, AFD, ACORD, SIDA, IAC
	Grey literature	ZETOC

^a^ Pascal was mentioned in the protocol but was not accessible; ‘Solutions for public health’, BMJ Clinical Evidence and EBRD were searched but found not relevant – searches were discontinued

Searches retrieved documents referencing MDEs, LMICs and procurement. Two independent reviewers (KD and SB) screened titles for relevance, discarding documents not referencing MDEs. KD and MB further screened abstracts according to pre-specified inclusion/exclusion criteria (Fig. 1). We retained documents with explicit references to MDE procurement processes or procedures within LMICs and excluded material focusing on the procurement/evaluation of a single device or solely on LMIC medical technology regulatory issues. Disagreements on the inclusion/exclusion of studies involved consultation of a third reviewer (SB) and were resolved by consensus.

### Data collection

One reviewer (KD or MB) extracted data on a pre-specified list of questions from all included documents. (See protocol) Questions related to: normative or descriptive accounts of MDE procurement and technology management processes; the relevance of health technology assessment exercises and health needs assessments in procurement; the input of health care professionals or specialist staff (e.g. biomedical engineers, economists) in procurement decisions; device installation, maintenance and decommissioning procedures/recommendations; health service delivery levels and clinical guideline procurement recommendations; budget impact, technology costs and intended national/regional coverage levels. We ascertained if documents included explicit accounts of MDE prioritization processes and extracted quotations or descriptions of processes for qualitative analysis.

### Analysis

We employed two methods of analysis to summarize and interpret data extracted. (Additional file 2) Narrative synthesis was used to offer a summative and descriptive overview of all included documents for issues relevant to research questions posed [15]. Qualitative meta-summary was used to explore MDE prioritization for a subset of documents outlining explicit prioritization methods/processes. We iteratively applied descriptive codes to the extracted data and then grouped similar codes into categories and themes; effect sizes are calculated as per Sandelowski et al. and indicate the % of documents citing a specific theme. We explored emergent patterns and relationships between themes to arrive at summative findings [16].

### Reporting

We follow PRISMA reporting guidelines as applicable – See Additional file 3 for a PRISMA checklist [14, 17].

### Role of funding source

The funder of the study had no role in study design, data collection, analysis and interpretation or writing of the report. The corresponding authors have full access to all the data in the study and had final responsibility for the decision to submit for publication.

## Results

### Bibliometric analysis

**Fig. 2:**
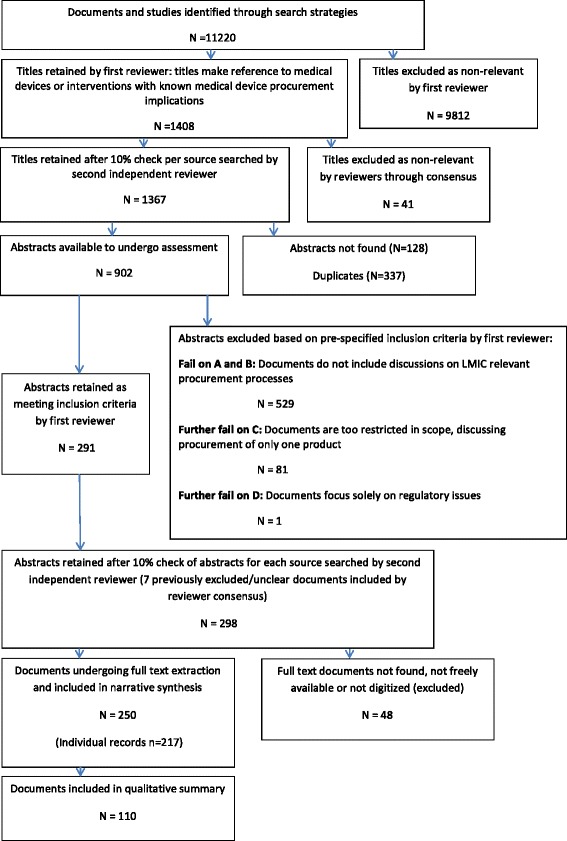
PRISMA Flowchart

Our search strategy located 11,220 unique documents of which we selected 217 for inclusion in the systematic review, all published 1984–2013. As several documents retrieved were entire books or journal issues where more than one chapter or article met our inclusion criteria, we extracted data from 250 individual full-texts. Figure 2 shows a PRISMA flow-chart outlining the study selection process and Additional file 4 includes a full reference list of included documents (marked SR# in Tables), ancillary characteristics and data extracted.

Tables 2 and 3 present characteristics of documents included. The majority are peer-reviewed journal articles (*n* = 125, 50%) and recommendations or guideline documents. (*n* = 72, 29%) The WHO and other UN associated organizations authored 141 (56%) documents. Only 50 (20%) documents refer to specific countries or regions, the remaining documents referencing resource poor settings or LMICs in general (Table 3).

**Table 2:** Types of documents included in the systematic review and type of issuing organization

	Research institutions or academic groups	LMIC national health authorities	International consultants, NGOs or public health monitoring organizations	Hospitals or health care delivery facilities	Medical device manufacturers	Government sponsored donor organizations and the WorldBank	WHO and UN associate	Not identified	Total number of documents (% of total)
Article	47	8	25	16	2		25	2	125 (50%)
Guideline	1	2	6				25		34 (14%)
Manual							14		14 (6%)
Procurement notice		10	1						11 (4%)
Recommendation	4	1	5	3		5	19	1	38 (15%)
Report						1	6		7 (3%)
Other		3	3	1		2	12		21 (8%)
Total number of documents (% of total)	52 (21%)	24 (10%)	40 (16%)	20 (8%)	2 (1%)	8 (3%)	101 (40%)	3 (1%)	250

Definitions: Research institutions or academic groups = Universities, specialist research bodies or collaborations; LMIC national health authorities = national governments, government units or departments; International consultants, NGOs or public health monitoring organizations = Organizations such as Management Sciences for Health, the Centre for Disease Control among others; Hospitals or health care delivery facilities = organizations with clinical health service delivery remit; Medical device manufacturers = commercial entities and device suppliers; Government sponsored donor organizations and the World Bank = USAID, DFiD, GIZ, CIDA and the WB; WHO and UN Associate = WHO, PAHO and UNDP, UNFPA, UNAIDS; Not identified = document authors solely, no identified issuing organization

Article = peer-reviewed material published in academic journal or magazine; Bulletin = notification; Presentation = conference presentation or talk/speech; Guideline = document identifying guiding principles and procedures; Procurement notice = tendering or bidding documents, initial advertisements of tender; Recommendations = Research or review documents providing clearly stated summary recommendations; Report = document with pre-specified topic, may include research evidence, discussion of current and best practice; Other = consultative document, evaluation/audit document, information booklets, policies, resolutions, databases or spreadsheets, websites

**Table 3:** Particular countries and regions referenced in documents included ^a^ (frequencies of citation) grouped according to 2014 World Bank Country classification

Category	Country/region (frequency of citation)
Low-income countries	Benin (1), Guinea-Bissau (1), Congo (1), Mali (1), Chad (1), Eritrea (1), Ethiopia (2), Gambia (1), Afghanistan (2), Bangladesh (2), Kenya (1), Malawi (1), Morocco (1), Nepal (3), Tanzania (3), Uganda (1), Zimbabwe (1)
Lower-middle-income countries	Bolivia (1), Cameroon (1), Guyana (1), Mongolia (1), Pakistan (1), Philippines (1), Vietnam, (1), Zambia (1), Lesotho (1)
Upper-middle-income countries	South Africa (1), Peru (1), Brazil (3), China (2), Thailand (1), Mexico (1)
High-income countries	Chile (1) ^b^, USA (1) ^c^
Regions	Balkan countries (1), Eastern Europe (2), Africa (1)

^a^ Citations are made in 50 documents (one document may refer to more than one country) Remaining documents reference LMICs generally

^b^ Chile was classified as an upper middle-income country up to 2014

^c^ The USA is used as a comparator in one study

As procurement methods may differ by technology, we extracted data on cited health conditions/clinical interventions (Table 4) and MDE descriptions (Additional file 5). Predominantly, documents reference HIV/AIDS and associated comorbidities (*n* = 29, 12%) and interventions for reproductive, maternal and child health. (*n* = 23, 9%) MDEs cited include laboratory devices (*n* = 22, 9%), equipment for surgical care (*n* = 16, 6%) and reproductive health (*n* = 16, 6%). Various classification systems for equipment or devices were used, including categorization according to size, cost, clinical area or health service delivery level (Additional file 5).

**Table 4:** Specific health conditions, disease areas and services/interventions cited across the included literature (frequencies of citation) ^a^

Health conditions and disease areas cited and frequency of citations		Service areas/interventions cited and frequency of citations	
AIDS/HIV and associated comorbidities	29	Interventions for reproductive, maternal and child health	23
Cancer	16	Surgery and trauma care	13
High burden diseases: diarrhoea, malaria, HIV, respiratory issues	7	Emergency medicine and disaster response	4
Malaria	5	Injection practices	2
Cardiological conditions	3	Imaging	2
Respiratory conditions, asthma and COPD	3	Blood safety; Forensic science; Primary care	1 each
Tropical diseases	2		
Gastroenterological conditions	2		
Tuberculosis	2		
Bacteriological diseases and interventions; Measles; H1N1, H5N1; Narcotic use; Renal disease; Non-communicable diseases; Fractures and orthopaedic conditions; Cardiovascular disease	1 each		

^a^ Total *n* = 124, remaining documents do not include references to specific health conditions. (One document may reference more than one condition/clinical area)

### Procurement structures and relevant stakeholders

We distinguish descriptions of MDE procurement structures – i.e. how stakeholders interact and reach decisions, from procurement methods – i.e. algorithms or approaches used to determine which technologies to purchase.

**Fig. 3:**
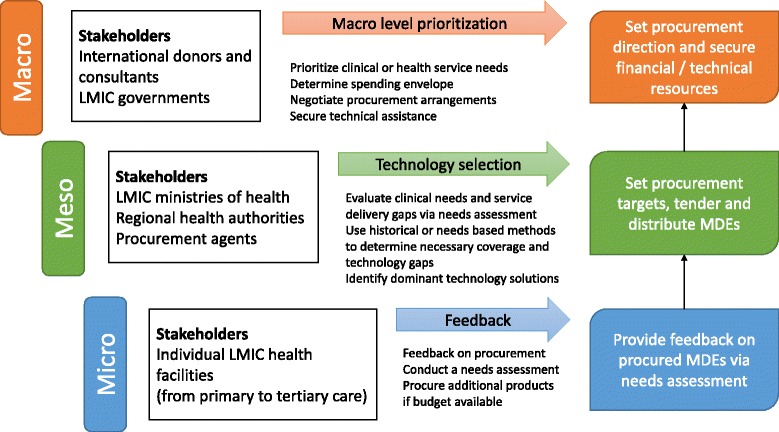
Stakeholders and MDE procurement planning steps by health system level*. *The above diagram was developed following narrative synthesis and qualitative summary of all available data; diagrams are descriptive accounts of the literature. Stakeholders are grouped by the health system level at which they operate and the MDE procurement planning actions stakeholders undertake are indicated. The coloured boxes on the right represent summary goals that stakeholders at each level intend to meet in relation to MDE procurement

Appraised documents identify different stakeholder groups interacting to reach procurement decisions; stakeholders range from international donor agencies, LMIC governments and ministries of health to individual LMIC health facilities. We classify stakeholders descriptively according to the health system level at which they operate and provide a summary of their attributed roles in Fig. 3. We note that procurement activities frequently involve all stakeholder groups outlined; we identified only one document where donors solely undertook procurement activities on behalf of LMICs [18].

At macro level, international donor agencies and LMIC governments engage in procurement partnerships. LMICs possess the human resource and health system capacity to support donor campaigns; in turn, donors share financial and technical resources. For example, Management Sciences for Health (on behalf of USAID) prompted the government in Afghanistan to use health economic and ethical criteria in defining the basic and hospital care package [19, 20]. Donors (e.g. USAID) and international agencies (e.g. UNICEF) enjoy a greater share of market power than LMICs due to their involvement in multi-country procurement. Donors thus provide an advantageous negotiation position for LMICs, helping secure flexible payment or bulk-pricing arrangements [21, 22].

Potential disadvantages of donor involvement include sudden discontinuation of assistance arrangements and restrictions on financial aid [22]. For example, donors may restrict financing to countries adhering to strict procurement/tendering regulations; such restrictions may preclude LMIC governments from strengthening technology-manufacturing capacity through the award of national procurement contracts [21]. Similarly, funding opportunities may be restricted to donor-preferred causes such as HIV/AIDS diagnosis and treatment, and preclude investments into incipient health system infrastructures, including for example sanitary provisions (e.g. water and sewage), electricity supply and infection prevention and control protocols [23].

At meso level LMIC governments, their ministries of health and relevant subunits engage in the minutiae of acquisition planning, tendering and equipment distribution/oversight activities. Stakeholders set procurement targets - i.e. project what equipment to procure via the use of experience or needs based planning methods (see next section) - and agree national technology distribution plans [1, 24].

Not all medical device procurement decisions are made at regional, country or supra-national level: individual health facilities also engage in direct acquisition [25, 26]. Authors of reviewed documents caution that such practices are not consistent across LMICs: hospitals frequently lack dedicated funding for MDE procurement and may instead rely on donations, reuse and recycling practices, to meet technological needs [25, 27, 28].

Whichever stakeholders engage in procurement processes, we note the literature is largely unclear on how stakeholder views are aggregated or divergent opinions handled - we have identified only three documents including descriptions of such accounts. Nobre et al. point to the usefulness of multi-criteria decision analysis methods, aimed to aggregate and integrate individual decision-makers opinions [29]. Using this method, decision-makers involved in the procurement of MDEs as well as clinical or financial administration staff score technologies on a relevant and clearly defined set of criteria – e.g. benefit to the patient population. The highest scoring technologies are then procured. Such processes may, however, be inherently biased: the experience of decision-makers may not in fact reflect best available evidence globally.

Consensus methods or DELPHI processes (recognized as particularly labour-, skill- and resource-intensive) [30] could be used. For example, considering the use of cryotherapy for cervical intra-epithelial neoplasia, the WHO commissioned independent systematic reviews of priority topics relating to cervical neoplasia in women, selected a panel of 14 multidisciplinary experts to review developed GRADE evidence profiles and chaired a meeting during which experts reached a consensus on key recommendations on the topic, including technology use.

Health technology assessment methods and routine committee based evaluations of procurement processes [31] may be employed. PAHO recommends multi-disciplinary committees involved in MDE procurement draw on the evidence compiled by either national or regional HTA bodies to reach purchasing decisions.

### Procurement planning methods

Two main methods for MDE procurement planning in LMICs were described in included documents. Firstly, stakeholders may rely on experience to determine what equipment to procure: e.g. past procurement and consumption patterns are reviewed and used as a template for reaching current and future decisions. For example, this method may be used to keep an existing laboratory functional provided service delivery does not change [24].

Secondly, in contrast to experience-based methods, needs-based procurement relies on stakeholders identifying explicit health priorities at any given time and agreeing service delivery targets based on context specific epidemiological information. For example, the WHO Priority Medical Devices Availability Matrix identifies conditions corresponding to the highest global (or national) burden of disease and indexes interventions corresponding to these conditions [32]. Devices necessary for carrying out each intervention are listed and added to a ‘wish list’. Such methods thus identify prescient health needs and evaluate procurement options in the context of defined vertical/horizontal programs, available budgets, present physical infrastructure and human resource skill mix/availability [33–35]. Needs-based methods may also rely on the development of basic or advanced health care packages- e.g. see the Basic Package for Primary Care Services by the Ministry of Health in Afghanistan [19, 20].

In practice, stakeholders are reported to use mixed approaches. For example, CENETEC in Mexico uses historical procurement trends to recommend what equipment to buy in clinical areas with little to no innovative or updated practice, and needs-based methods to issue procurement recommendations for national priority health care areas such as tele-medicine or cancer care [36].

### Factors considered in procurement planning

**Fig. 4:**
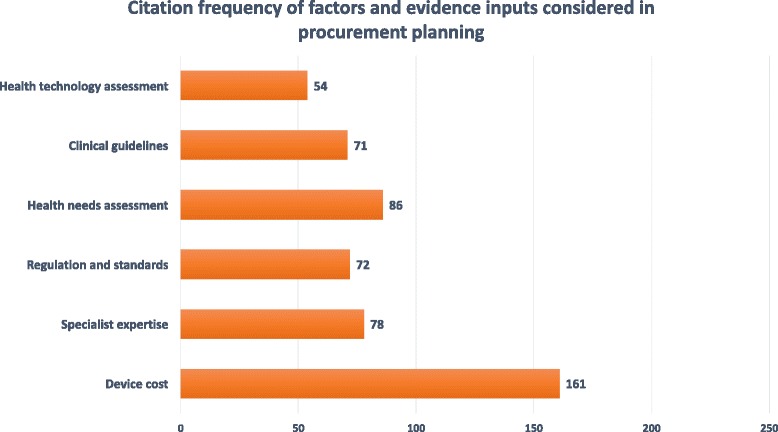
Citation frequency of factors and evidence inputs considered in procurement planning

We distinguish factors considered in procurement planning from factors affecting the successful deployment and use of MDEs in health facilities (See next section). Figure 4 provides a visual representation of factors, including evidence inputs, stakeholders consider when reaching procurement decisions; we note the citation frequency of each item and suggest this as a proxy for the relative importance of the factor in decision-making. Table 5 further summarizes frequent challenges and best practices encountered for each of the above factors and inputs.

**Table 5:** Evidence inputs and factors considered in medical device procurement planning

Factors/ Evidence input	Areas of concern in current procurement planning processes	Selected key references ^a^	Recommended course of action to address areas of concerns / best practices:	Selected key references ^a^
Medical device cost: costs considered for each product purchase	Installation, maintenance and safe disposal costs not captured; User training costs not included;	SR143: WHO, 2011 SR122, 124–131: WHO, 2010	Include all expenses associated with medical device deployment to health facilities, in particular user training and maintenance;	SR241: Martin, 2005 SR247: Free, 1993 SR122, 124–131: WHO, 2010
Specialist expertise: advice or opinion of biomedical engineers, health economists, clinical or procurement specialists considered when planning	Experts are rarely locally available; Where experts are available, expertise is likely financing/ pharmaceutical rather than device specific;	SR79: Mullally, 2008 SR26: Mundy, 2012 SR34: Mundy, 2012	If possible, create national training programs/specialized procurement units staffed with biomedical engineers; Consult international biomedical engineers or health economists on specifications and value for money of products;	SR63–69: Bloom, 1989 SR80: Mytton, 2010
Regulations and standards: Equipment conformity to international regulatory approval (FDA approval, ISO certification, CE mark)	Products complying with international regulatory approvals may be costly and unavailable in local markets; Absence of national regulatory agencies impedes verification of certifications;	SR163: WHO, 2012 SR133: WHO, 2011	As a minimum standard, ensure high-risk equipment is internationally certified for use so as to ensure patient safety;	SR35: Keller, 2010
Health needs assessment: Identified population health priorities and/or technological needs	National level decision-makers may distrust needs-assessments conducted by health facility personnel due to exaggerations or mis-information; Needs assessment information may not be up to date;	SR38: Aid-Khalet, 2001 SR56: Mavalankar, 2004	Create regional or national level participatory structures where health facility representatives may directly participate in procurement planning and tendering.	SR122, 124–131: WHO, 2010 SR176: WHO, 2000
Clinical guidelines: Patient management guidelines for interventions/clinical areas	Clinical guidelines may not include information on products needed to carry out specific health interventions;	SR184: Anderson, 2008	Incorporate indications on medical device necessities into clinical guidelines and where possible advise on LMIC friendly product specifications.	SR41: Briggs, 2008 SR44: Dyer, 2010
Health technology assessment: Methods and reports on the procurement economic and health impact, and policy and regulatory approval	HTA difficult to undertake due to data paucity on health impacts, medical device coverage, equipment life span, true costs of equipment.	SR24: PAHO, 2012 SR249: Withanachchi, 2007	Within resource constraint, adopt transparent and evidence-based processes to evaluate different investment options; If possible, secure political support for HTA implementation.	SR106: Panerai, 1989 SR198: Teerawattananon, 2005

^a^ Numbers with a prefix “SR” shown in the Table refer to identification numbers for documents included in the systematic review – please see Additional file 4 to identify the individual references

Equipment cost, specialist recommendations and technology regulatory approval are the primary factors reported as influencing procurement decisions. Authors of reviewed documents caution that procurement stakeholders underestimate the true cost of MDEs as they neglect to consider maintenance, servicing and user training requirements [1, 36–41]. Across the literature, the input of specialists is recommended to ensure improved technology procurement: e.g. biomedical engineers can advise on maintenance/servicing/user training issues, and health economists on the relative cost-effectiveness of technologies [1, 39, 42–48]. Appraised documents also cited international certification (e.g. approval by the FDA, a CE mark in the EU, inclusion in a WHO prequalification scheme) as a proxy for technology safety, a desired feature of MDEs to be procured [38, 49, 50].

Evidence inputs identified across the literature include: health needs assessment exercises/reports, clinical guidelines and health technology assessment exercises/reports. The former factor is cited in relation to needs-based procurement methods: i.e. routine health-needs appraisals clarify national investment priorities [32]. Authors of reviewed documents widely endorse the use of clinical guidelines for technology selection; however note these do not historically include clear technology investment/use recommendations [32]. We found MDE availability checklists and tools designed around clinical guidelines [51–53].

Included documents additionally assign importance to health technology assessment (HTA) exercises/reports [39, 46]. Such evidence inputs are mentioned infrequently, and when present, studied authors comment on the difficulty of undertaking HTA (health economics in particular) within resource constrained settings due to data paucity, lack of specialist capacity and funding, and a general lack of knowledge on how such evidence may feed into decision making processes [1, 47, 48]. MICs, however, have made substantial progress in the use of HTA for the promotion of transparent and evidence-based decision making: e.g. see HITAP in Thailand, CENETEC in Mexico and a bill for the promotion of HTA use across Latin America [31, 36, 47].

### Factors affecting device deployment

**Fig. 5:**
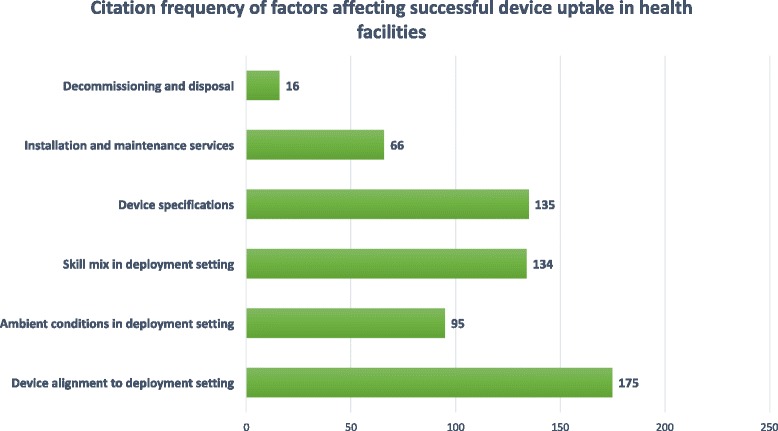
Citation frequency of factors affecting successful device uptake in health facilities

We also extracted data on the factors cited as affecting successful MDE uptake or use in LMICs. We provide a visual representation of factors and citation frequency in Fig. 5 and summarize frequent challenges and relevant best practices in Table 6. Authors emphasise that decision-makers must carefully consider MDE technical specifications and alignment to deployment setting infrastructure, as well as ambient conditions and skills mix encountered therein, before reaching a procurement decision. We provide a summary of MDE design characteristics most frequently mentioned by authors in Table 7 and suggest these as a starting point for specification of desired technology characteristics or product triage during procurement.

**Table 6:** Challenges affecting successful medical device uptake and use

Challenge	Consequences if challenge left unaddressed	Best practice
Device not aligned to healthcare delivery level and general conditions encountered in deployment setting (mix of skill mix, ambient conditions, referral pathways)	Device cannot be used or falls into premature disuse	Consult clinical guidelines/experts
Ambient conditions in deployment settings prevent the use of the device;	Product cannot be used; Product does not reach full life-expectancy;	Develop technological needs assessment: note present conditions; consult LMIC friendly specification list
Health care personnel not trained in safe medical device use or maintenance: devices not used safely and do not reach full life-expectancy	Products used unsafely - patients may experience adverse health outcomes.	Provision of training manuals and supplier training for any purchase
Device specifications to accord to the conditions in which it is to be used: e.g. durability, humidity/temperature resistance	No clear indication of LMIC friendly device specifications	Device specifications should conform to LMIC environment and settings (see Table 7)
Installation and preventive and corrective maintenance services available (including necessary financing)	Lacking financial and human resources to carry out maintenance/servicing of available devices	Installation and maintenance services should be included as part of medical device procurement and all ancillary costs considered in procurement
Provision for safe medical device decommissioning and disposal; including financial resources	Lacking financial and human resources to carry out	Identify decommissioning or disposal mechanisms and consider any cost implications

**Table 7:** Medical device specifications and design desirable for LMIC settings ^a^

Design domain	Specification
User friendliness	Easy to use; rapid; low training needs
Portability	Compact and portable (choose desktop variety if theft is an issue) Avoidance of bulky and heavy design
Reliance on external factors	Elimination of external power sources Include water purification system Minimal need for sample preparation Minimal need for spare parts
Design	Long shelf-life at ambient temperature Rapid High sensitivity and specificity for diagnostic technology High throughput
Material	Robust Choice of durable material

^a^ The above design characteristics were identified following thematic analysis and coding of documents included in the qualitative meta-summary

We note that factors cited as affecting MDE use in deployment settings relate closely to the issues that authors of reviewed documents suggest should be considered during procurement planning: e.g. specialist expertise – in the form of both biomedical engineering and clinical knowledge – is needed to advise on what products are suitable given a health facilities’ ambient conditions and intended service delivery program. Similarly, a product’s total life-cycle cost is highly dependent upon the installation, maintenance, decommissioning and disposal services that need to be put in place to support product deployment; should human resource training be needed this will additionally carry financial implications.

### Prioritization of MDEs for procurement

One hundred and eleven of the 250 reviewed documents indicate specific MDE prioritization methods and were included in the meta-summary. Please see Additional file 6 for a full account of findings generated and codes and abstracted themes/topics developed during the meta-summary; we restrict our discussion here to prioritization criteria.

**Fig. 6:**
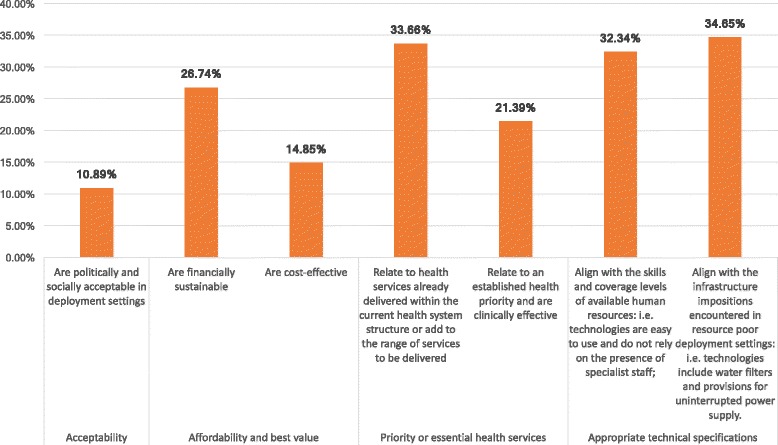
Prioritization criteria extracted from reviewed literature (% indicate meta-summary effect size)*. *The above criteria were distilled from the meta-summary presented in Additional file 6. Criteria relate to the thematic analysis conducted across the 111 documents noting explicit MDE prioritization processes. Effect sizes are calculated as per Sandelowski et al. and indicate the % of documents citing a specific theme

We identify six main prioritization criteria across reviewed documents, which correspond to both normative and feasibility conditions, and list these in Fig. 6 according to relative importance assigned in the literature. Recurrent themes in the literature concern the identification of priority health areas and services as well as the identification of technologies suitable to deployment settings (See Table 7). For example, the WHO, USAID and UNFPA all recommend prioritizing MDEs used in interventions addressing prominent disease burdens and that support existing health service delivery efforts [27, 32, 54]. Purchases are further screened and prioritized according to their suitability to LMIC settings: i.e. MDEs for which no trained professionals are present or which lack established maintenance or decommissioning services are deprioritized for purchase [28, 36, 38, 55–57].

Budget constraints, experiences gained in past procurement cycles, political/cultural support and equity considerations also influence prioritization decisions. Current and future budget impact is balanced against evaluations of past procurement performance: e.g. if supply chains are not present to source a particular technology, this is either deprioritized or alternative sources for investment identified [58]. Non-invasive, culturally acceptable technologies with records of accomplishment and safe use are preferred; however, in practice, technologies endorsed by political groups may further bypass normal prioritization or decision-making channels and be procured due to strong advocacy [59].

**Fig. 7:**
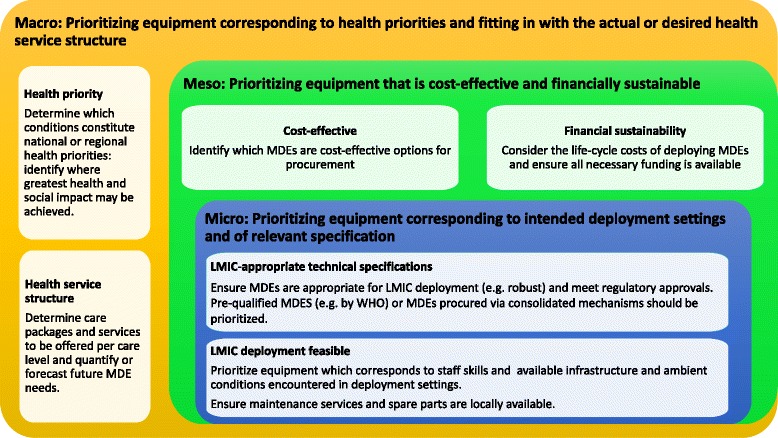
Medical device prioritization: decision-making issues and criteria considered at different health system levels*. *Following on from the qualitative meta-summary, we grouped the decision-making issues and criteria identified via thematic analysis by health system level. Issues and criteria are descriptive findings synthesized across documents

Patterns in extracted texts suggest different types of criteria are considered at different decision making-levels (Fig. 7). We interpret this as stakeholders at each health-system level undertaking/being responsible for different prioritization steps. For example, micro-level stakeholders - i.e. health care professionals in individual health facilities - prioritize equipment according to technical specifications and design: portable, durable, electric-surge resistant equipment is preferred [60, 61]. Meso-level stakeholder - i.e. regional and specialist long-term use, and ideally cost-effective [35, 62]. This meta-summary is descriptive in relationship to health system levels and roles but presents normative criteria although the descriptive and normative were not always clearly delineated in the source documents.

### Distilled recommendations

Table 8 provides a descriptive synthesis of normative MDE procurement and prioritization accounts reviewed. All findings reported are descriptive in that we synthesize theories and positions articulated within the reviewed literature: as part of this literature includes normative accounts, we have additionally synthesized these for those documents reporting specifically on prioritization of MDEs for procurement. We synthesise key recommendations and best practices and offer these for the consideration of procurement officials and MDE researchers.

**Table 8:** A synthesis of recommendations expressed in the literature for consideration by international donors, LMIC stakeholders and the international research community ^a^

Recommendation	Explanation
Close the feedback loop	The WHO deplores the mismatch created by low-resource settings procuring high-end technologies. (1) Authors in the literature recommend LMICs and donor institutions evaluate past procurement efforts and create participatory structures for health facility representatives to engage in planning/procurement consultations. This increases transparency and pre-empts technology adoption/use issues by informing all stakeholders of health facility needs/infrastructure/skill mix.
Fully cost out potential purchases	Authors in the literature note discrepancies in costing practices, we therefore recommend national costing templates are created and disseminated to facilities and procurement agents for MDE purchases. Costing templates should be context specific and include: a) Expenses related to equipment installation, servicing (inspection, installation, preventive and corrective maintenance, decommissioning and disposal); b) Investments into infrastructural refurbishments of deployment health facilities and user training that would aid in keeping MDEs operational. The WHO Cost-It templates present a good starting point for this at hospital or program level. We remind users to include inspection, installation and decommissioning/disposal costs in templates under the ‘other’ headings. (75)
Make MDE servicing a legal requirement	Authors in the literature recommend LMIC regulatory agents develop minimum, legally binding, standards for national/regional MDE servicing. Procured equipment should be subject to specialist inspection and installation once in deployment settings; service provisions/funding allowances for preventive and corrective maintenance, decommissioning and disposal should be identified before tendering.
Include explicit MDE availability recommendations in clinical guidelines.	We note that historically clinical guidelines do not include specific recommendations on what medical devices should be available for specific interventions - authors note this as an issue for biomedical engineers or procurement agents engaged in product selection.
Develop a list of generic specifications for LMIC friendly equipment.	Authors in the literature recommend the elaboration and listing of generic medical device and equipment technical specifications to aid LMIC procurers. The list of broad product features we have identified in this review is a start in this endeavor, but international engineering expertise is needed to create technical specifications or target product profiles specific to LMICs (Table 7).

*Abbreviations*: *MDE* Medical devices and equipment, *LMICs* Low- and Middle-income Countries

^a^ This synthesis was developed following on from the narrative synthesis and meta-summary. It reflects the authors’ reflections on themes and issues emphasized in the literature

## Discussion and conclusions

The current paper is the first review to systematically appraise and summarize the LMIC medical device procurement literature. We acknowledge some limitations. First, the LMIC MDE specific procurement literature is highly heterogeneous; our search and selection algorithms were therefore deliberately broad. The review thus provides not only a synthesis of the available literature but also serves as a hypothesis generating exercise meant to direct future research efforts and inform current procurement professionals of key recommendations in the global literature. Second, when appraising and synthesising information across texts, it was not always possible to distinguish descriptive and normative MDE procurement accounts: i.e. what happens in practice vs. what ought to happen. Despite this, we offer readers a structured account of the reported methods, factors and prioritization criteria considered for MDE procurement.

The heterogeneity of documents reviewed, specifically the diverse bibliographic literature (e.g. ranging from cost-effectiveness evaluations to guidance on the use of health technology assessments methods for procurement) and large proportion of grey literature included in this review, made assessments of the risk of bias impractical. We acknowledge this as a limitation and caution readers to reflect on findings carefully.

We acknowledge substantial difficulties in sourcing documents for full-text review. While we appraised several digitized abstracts, it was not always possible to locate ancillary digital full-text versions of documents of interest: while we are confident that these documents cannot be openly accessed, we were unable to assess any bias associated with availability. We additionally note that our findings reflect the state of the literature up to 2013. Further documents of relevance to MDE procurement have been published in recent years (e.g. [63, 64]), however to our knowledge, such documents represent updates or continuation of literature included here rather than research into the specific MDE procurement methods relevant for LMICs.

The comprehensive synthesis of information, as well as the granular pragmatic recommendations distilled across documents, are the principal strengths of this systematic review. In our narrative synthesis we identify two general methods for MDE procurement planning, which stakeholders appear to combine in practice: experience-based methods rely on the perceived success of previous purchase rounds; need-based methods instead identify current health needs and develop bespoke technology procurement plans to tackle these. Overall, we find no established consensus on how LMIC based MDE procurement should occur, but we note the literature converging on what evidence inputs and factors should be considered in decision-making.

The findings of the meta-summary echo previous work on the normative and feasibility criteria considered by decision makers in technology investment and prioritization [65–68]. In contrast to previous conclusions emphasizing the relevance of normative criteria, however, we note that MDE procurement is chiefly driven by feasibility concerns: i.e. as MDEs run the substantial risk of being unused due to technology misalignment to deployment settings, decision makers most value products with appropriate technical specifications and established maintenance services. We recommend further research be undertaken to support the development and validation of a unified set of criteria able to guide LMIC medical device and equipment procurement. Criteria identified within this paper may serve as a first draft of such a document. Further research may additionally explore differences between international and national guidelines on the topic, as well as national guidelines and sub-national practices.

Our findings further suggest that MDE procurement within LMICs presents substantial differences to technology procurement within HICs. While individual health facilities may have the capacity to directly tender in the latter settings, we have noted this practice is not consistent across LMICs. HICs further use health technology assessment agencies and health economic principles and methods to select technologies appropriate for reimbursement and advise on the containment of health care costs [69, 70]. In this review, only a fifth of documents reference such methods for MDE procurement. Difficulties in using such methods for LMICs are widely noted in the literature and have more recently been summarized in the 2015 Global Survey on Health Technology Assessment; political, cultural and specialist support for the use of such methods is lacking and the necessary data on local epidemiology, costs and treatment impact for LMICs is also scarce [71]. We contend, however, that such efforts are recommended for the development of transparent and rational procurement practices [68, 72, 73]. We developed a decision algorithm incorporating health economic methods that may be suitable for LICs with little specialist capacity elsewhere [74] and direct readers to further valuable resources on this topic [68, 73, 75].

## Additional files

Additional file 1: Appendix 1: Example search strategy for MEDLINE (OVID SP). (DOCX 14 kb)

Additional file 2: Appendix 2: Search and selection algorithm. (DOCX 27 kb)

Additional file 3: Appendix 3: PRISMA 2009 checklist. (DOC 62 kb)

Additional file 4: Appendix 4: Data extraction sheet. (XLSX 85 kb)

Additional file 5: Appendix 5: Equipment categories as noted in the reviewed literature (*n* = 131). (DOCX 16 kb)

Additional file 6: Appendix 6: Findings of the qualitative meta-synthesis and supporting coding structure. (DOCX 23 kb)
