# Supplementary material for: Methods for medical device and equipment procurement and prioritization within low- and middle-income countries: findings of a systematic literature review
Source: Global Health. 2017 Aug 18;13:59. doi: 10.1186/s12992-017-0280-2 (PMC5563028; doi:10.1186/s12992-017-0280-2)
Supplement: Supplementary file 1 — Appendix 1: Example search strategy for MEDLINE (OVID SP). (DOCX 14 kb) [file 12992_2017_280_MOESM1_ESM.docx]

**Appendix 1:** **Example search strategy for MEDLINE (OVID SP)**

| 1. device.mp. or exp "Equipment and Supplies"/  2. (device* or equipment* or suppl*).mp. [mp=title, abstract, original title, name of substance word, subject heading word, keyword heading word, protocol supplementary concept, rare disease supplementary concept, unique identifier]  3. exp Technology, Radiologic/ or exp Technology Assessment, Biomedical/ or exp Fiber Optic Technology/ or exp Educational Technology/ or exp Biomedical Technology/ or technology.mp. or exp "United States Office of Technology Assessment"/ or exp Technology/ or exp Food Technology/ or exp Technology, High-Cost/ or exp Technology Transfer/ or exp "National Center for Health Care Technology (U.S.)"/ or exp Wireless Technology/ or exp Technology, Dental/ or exp Green Chemistry Technology/ or exp Technology, Pharmaceutical/ or exp Remote Sensing Technology/  4. 1 or 2 or 3  5. (procure* or purchas* or acqui* or commission* or buy*or order*).mp. [mp=title, abstract, original title, name of substance word, subject heading word, keyword heading word, protocol supplementary concept, rare disease supplementary concept, unique identifier]  6. (countr* adj2 (income or poor or poverty or develop* or resource or low* or mid*)).mp. [mp=title, abstract, original title, name of substance word, subject heading word, keyword heading word, protocol supplementary concept, rare disease supplementary concept, unique identifier]  7. (third adj2 world).mp. [mp=title, abstract, original title, name of substance word, subject heading word, keyword heading word, protocol supplementary concept, rare disease supplementary concept, unique identifier]  8. (emerging adj2 (econom* or market*)).mp. [mp=title, abstract, original title, name of substance word, subject heading word, keyword heading word, protocol supplementary concept, rare disease supplementary concept, unique identifier]  9. developing country.mp. or exp Developing Countries/  10. 6 or 7 or 8 or 9  11. 4 and 5 and 10  Conducted last: 28 January 2013, 15:10 (GMT)  Number of records identified: 2297 |
| --- |
