# Supplementary material for: Methods for medical device and equipment procurement and prioritization within low- and middle-income countries: findings of a systematic literature review
Source: Global Health. 2017 Aug 18;13:59. doi: 10.1186/s12992-017-0280-2 (PMC5563028; doi:10.1186/s12992-017-0280-2)
Supplement: Supplementary file 2 — Appendix 2: Search and selection algorithm. (DOCX 27 kb) [file 12992_2017_280_MOESM2_ESM.docx]

All remaining documents eligible for inclusion in review and undergoing data extraction provided full text documents are digitized and freely available

Documents retrieved through search strategy

(Retain digitized materials with abstracts available)

Selection Step 1:

Title appraisal (One reviewer)

Retain: all titles referencing medical devices

Selection Step 2:

Title appraisal (Second independent reviewer)

Random 10% check of titles per each source searched

Selection Step 3:

Abstract appraisal (One reviewer)

See Figure 2 for selection algorithm

Selection Step 4:

Abstract appraisal (Second independent reviewer)

Random 10% check of abstracts per each source searched

Selection Step 5

(Optional: to be used when abstracts prove ambiguous)

Full-text appraisal: Use criteria for abstract review

**Appendix 2: Search and selection algorithm**
