# Supplementary material for: Methods for medical device and equipment procurement and prioritization within low- and middle-income countries: findings of a systematic literature review
Source: Global Health. 2017 Aug 18;13:59. doi: 10.1186/s12992-017-0280-2 (PMC5563028; doi:10.1186/s12992-017-0280-2)
Supplement: Supplementary file 5 — Appendix 5: Equipment categories as noted in the reviewed literature (n = 131). (DOCX 16 kb) [file 12992_2017_280_MOESM5_ESM.docx]

**Appendix 5: Equipment categories as noted in the reviewed literature (n=131)**

| Classification of equipment (frequency of citation) | | Equipment or device cited (frequency of citation) | Selected key references* |
| --- | --- | --- | --- |
| Cost and size | High cost (>$25,000) (1)  Large medical equipment (1) |  | SR20: Nah, 2007  SR78: Miao, 2007 |
| Risk associated with use | High risk: implants (1) |  | SR 35: Keller, 2010 |
| Area of use | Surgical care and trauma  (16) and emergency care (7)  Reproductive, maternal and child health (16)  Cancer treatment (5)  Diagnosis (27)  Gastroenterology (1)  Respiratory (4)  Cardiology (4)  Ophthalmic (1)  Orthopaedic (1)  Cold chain, blood supply and transfusion services (9)  Infectious diseases (11) | Anaesthetic (5) Instrumentation and other devices (7) Oxygen supply and monitoring: concentrators and pulse oximeter (3) Intensive care (1) Instrumentation and resuscitation equipment (7)  Condoms and contraceptives (6) Birth kits and instrumentation (8) Obstetric instrumentation and devices (2)  Radiotherapy: megavoltage, linear accelerator (5)  Laboratory and RDT (16) Imaging and laboratory (6), CT and ultrasound (4), X-ray (1)  Gastroenterological equipment (1)  Ventilators, nebulizer, equipment for diagnosis of COPD (1), or asthma diagnosis/monitoring (4)  Biotechnologies (1) Refrigeration, injections, transfusion devices and storage (3)  Cold chain (2) Vaccines (1) Infection control (2)  HIV diagnosis and treatment (6)  Malaria diagnosis and treatment (2) Tuberculosis (3) | SR40: Arevalo, 2007  SR57: McCunn, 2010  SR60: Bewes, 1984  SR167: WHO, 2007  SR42: Chandani, 2001  SR82: Nessa, 1992  SR138: WHO, 1991  SR162: Borras, 1993  SR149: WHO, 2003  SR158: Palmer, 2011  SR84: Nicholls, 1984  SR197: IUaTBLD, 2008  SR15: Ribeiro, 2010  SR161: PAHO, 1999  SR71: Ruyter, 1984  SR19: Thorsteinsdottir, 2007  SR148: Lloyd, 1999  SR58: Ansa, 2002  SR256: Woodle, 2000  SR28: Walkowiak, 2008  SR90: Onwuwejke 2000  SR92: Parsons, 2011 |
| Health service delivery level | Primary (1)  Secondary or tertiary (9) | Primary level health care equipment(1)  Hospital: diagnostic and imaging, instrumentation (9) | SR141: Kaur, 2001  SR4: Unknown, 2005 |
| General descriptors | Miscellaneous (11) | Consumables/disposables, instruments, minor diagnostics and treatment/monitoring (8)  Waste management (1)  Injections (4) | SR18: Hussein, 2004  SR154: Pruss, 1999  SR117: Ekwueme, 2002 |

*References marked SR refer to documents included in the systematic review.
