# Supplementary material for: Methods for medical device and equipment procurement and prioritization within low- and middle-income countries: findings of a systematic literature review
Source: Global Health. 2017 Aug 18;13:59. doi: 10.1186/s12992-017-0280-2 (PMC5563028; doi:10.1186/s12992-017-0280-2)
Supplement: Supplementary file 6 — Appendix 6: Findings of the qualitative meta-synthesis and supporting coding structure. (DOCX 23 kb) [file 12992_2017_280_MOESM6_ESM.docx]

**Appendix table: Findings of the qualitative meta-synthesis and supporting coding structure**

| Findings of the meta-synthesis | Topic/ theme  (Effect size*) | Code | Definition | Effect size (%) | Example quotes or fragments  (Reference - See Appendix 2 for Reference List) |
| --- | --- | --- | --- | --- | --- |
| 1. Identification and understanding of health needs is a first step in identifying where greatest population impact can be achieved: relevant health care areas, including technology purchases, are prioritized.  2. Verification of health needs may play a role in restricting the procurement of medical devices: i.e. should devices and equipment not correspond to a priority health condition they should not be procured. | Identifying the priority health problems of a defined population in order to achieve health impact and benefits.  (ES: 21.39%) | General health condition | References to disease areas, issues or clinical guidelines without reference to these being a priority | 27.72 | “disease problem” (SR20)  “burden of disease” (SR26)  “from health problem to clinical guideline” (SR126) |
|  |  | Health needs assessment | Analyses of the health needs of a population, including epidemiological evidence | 32.67 | “certificate of need” (SR25)  “needs assessment (SR28)  “situational analysis” (SR59)  “local needs identified through prevalence and checked with providers” (SR142) |
|  |  | Achieving population benefits | References to how tackling a health problem (whether specified or not) yields benefits to populations | 14.85 | “benefit to the population, social impact, community and professional demand, importance for improving patient condition, expected benefits in health outcomes” (SR 87)  “treating and diagnosing TB is beneficial both for HIV and non-HIV patients”  “maximize use on different patient types” (SR 57)  “targeting health needs and adverse outcomes (risks)” (SR 132) |
|  |  | Health priorities | Discussion of health priorities or identified clinical areas/fields of priority | 9.9 | “clinical area to focus on: trauma care” (SR 220)  “prevalent emergency condition in reproductive health (SR 210)  “forensic science” (SR 234) |
|  |  | Achieving impact | Addressing health issues with the aim of achieving impact | 21.78 | “potential impact upon mortality reduction” (SR104) |
| 3. Cost-effective medical devices are prioritized for procurement.  4. Financing arrangements and constraints impact upon the choice of technologies for procurement:  i.e. if funding streams for particular conditions are available, devices for those conditions are prioritized within the funding round.  5. Devices and equipment imposing minimal costs upon the health system are prioritized for procurement. | Methods of intervention or technology evaluation  (ES: 14.85%) | Economic approaches to evaluation, including health economics | References to economic methods of evaluation to inform decision making | 27.72 | “establish cost-effective way of dealing with disease problem” (SR 20)  “cost-efficiency and effectiveness”( SR 159) |
|  |  | Health technology assessments for evaluation | Methods beyond economic evaluation, including consideration of needs, political support and other value considerations | 7.92 | “health technology assessment” (26)  “based on technology assessment and a six element approach: (…) boundaries and constraints, performance measures and measurement of actual performance” (SR 97) |
|  | Defining financial boundaries and seeking cost-minimizing solutions  (ES: 18.32%) | Financial constraints or thresholds | Budget constraints or thresholds set for equipment | 15.84 | “depending upon a cost-threshold a certificate of need process is adhered to” (SR 25) |
|  |  | Cost-reduction | Mentions of approach to minimize costs associated with technology purchases | 20.79 | “reduce cost” (SR 42)  “average and reasonable cost” (SR 45)  “ongoing costs” (SR 57)  “bulk purchases lead to cost reductions” (SR 155) |
|  | Exploring feasibility of purchases by defining financing arrangements and potential impact  (ES: 8.42%) | Linking impact with financial feasibility | Compound mention of impact and technological effectiveness | 9.9 | “impact, effectiveness, scalability” (SR 31)  “usefulness” (SR 58) |
|  |  | Financing arrangements | Financing sources for device procurement / management | 6.93 | “procurement linked to aid contracts and programs chosen by donors… diversion of money into different programs is unfavourable” (SR 55)  “financing ability” (SR 82) |
| 5. Consensus decision making methods and evaluations of past procurement processes and outcomes are preferred for reaching medical device and equipment prioritization decisions. | Methods for evidence evaluation and reaching medical device/technology selection decisions  (ES: 3.33%) | Procedural evaluations | Evaluations of processes related to procurement, including planning and further management | 3.96 | “Evaluation of technologies may not be sufficient, review the entire process: what do we want to achieve and how can it best be done?” (SR 163) |
|  |  | Consensus methods | Methods of reaching agreement regarding medical device procurement | 4.95 | “consensus method (involving experts) but focused on the review of systematic evidence” (SR 193) |
|  |  | Multi criteria decision making | Mathematical method of aggregating judgments on predetermined criteria | 0.99 |  |
| 6. Health care services and packages to be provided at each health care level directly influence which devices and equipment are prioritized and procured.  8. For newly introduced health services, policies for medical device management should be put in place. | Defining the health service structure  (ES: 33.66%) | Care packages and services to be provided at different health system levels | Determination of interventions and services to be provided at primary, secondary or tertiary care levels | 43.56 | “defining basic packages of care at each service delivery level” (SR 31)  “Collaborate with provincial and national authorities to find the suitable package for the setting” (SR 56)  “distance from clinical sites and structuring of services” (SR 89) |
|  |  | Defining services and procurement plans by defining targets and ensuring forecasting is done | Setting procurement targets and planning according to population/service use forecasts | 23.76 | “ensuring adjustment to patient volume” (SR 42)  “create purchasing plans with projections of use” (SR 63)  “ability to deliver pre-specifies treatment targets” (SR 75) |
|  | Creating policies for medical device procurement and management  (ES: 9.9%) | Creating policies and frameworks to address prioritization and device management | Mention of policies or management frameworks relating to medical devices and their procurement | 9.9 | “plan for a national policy on injection equipment and safety boxes” (SR 155)  “waste management policies should include details on where disposal happens in facilities, and whether disposal is regional or national” (SR 156)  “Prioritization is included as part of a health management policy but not with specific detail as to how to undertake this” (SR 228) |
| 9. Prioritize equipment which can be safely used and managed in deployment settings.  i) Prioritize equipment with LMIC friendly specifications.  ii) Prioritize equipment that can be easily used and maintained within health facilities. | Desired features of medical devices for LMICs  (ES: 28.71%) | Risk and safety | Associated risk of device use or misuse and issues of safety | 19.8 | “high risk devices” (SR 37)  “safety” (SR 31) and “safety profiles” (SR 82)  “variability and risk” (SR 178) |
|  |  | Device specifications | Desired or undesired characteristics of devices to be procured | 47.52 | “long-life” (SR 42)  “resistant to ambient conditions” (SR 56)  “electricity, device design (e.g. whether hand-held or desktop operated – theft may be an issue), weight, operating temperature and humidity, hard and robust casings, battery life, display type” (SR 57)  “high sensitivity and specificity” (SR 45)  “function and simplicity” (SR 109)  “devices are to compensate for lacking human resource skills and have reduced operational features” (SR 117) |
|  |  | Quality and standards | Reference to pre-qualification of products, and quality assurance and control procedures | 18.81 | “quality equipment” (SR 56) and “quality assured products” (SR 183)  “pre-qualification for the IUD showed one product suitable for most patients and manufacturing capacity was suitable to low-income settings as well” (SR 209) |
|  | Managing equipment in the field in LMICs: what is needed  (ES: 32.34%) | Matching facilities and their staff to medical device specifications | Convergence of facility design and conditions, staff abilities/training needs and medical device specifications in deployment setting | 49.5 | “facility type and conditions as well as experience of surgeon should dictate prioritization” (SR 62)  “skills and knowledge of staff” (SR 82)  “available local technical skills” (SR 89) |
|  |  | Maintenance and spare parts | Discussion of how maintenance should be conducted, why it is needed, how spare parts fit into the problem | 27.72 | “maintenance and service need priority and should be adapted to local needs” (SR 63)  “minimal parts and consumables, simple, minimal maintenance and expert input” (SR 165) |
|  |  | Supply of maintenance and spare parts | Who provides services and spare parts? | 19.8 | “local supplier availability for instrument maintenance and reagent supply” (SR 89) |
|  | Regulatory issues, approvals and surveillance processes  (ES: 5.94%) | Regulation | Specification of devices and how they accord with international or national regulatory frameworks | 5.94 | “ICF and ISO are used to delineate core sets of assistive products” (SR 127)  “prioritize health technology assessments at stages of pre-market clearance or post-market surveillance” (SR 128) |
| 10. Political, social and value considerations influence prioritization decisions.  11. Past procurement experiences influence current prioritization processes. | Procurement processes in LMICs: reports of relative successes  (ES: 10.89%) | Procurement processes in LMICs | Descriptions of how procurement processes work/do not work in LMICs | 10.89 | “Centralized procurement discouraged although may be advantageous, different timings of arrival observed” (SR 58)  “developing a rational and efficient tendering procedure involves not only price but also maintenance and service considerations, all should be adapted to local needs” (SR 63) |
|  | Political and social economy of procurement  (ES: 10.89%) | Political aspects of procurement | Mention of how politics shapes procurement | 7.92 | “Prioritization occurs around six factors: … (technology) to be politically responsible” (SR 88)  “Prioritization entails … the need for organizational sponsorship and development” (SR 104) |
|  |  | Value considerations in procurement | Account of which value judgments are incorporated in procurement | 13.86 | “Prioritization occurs around six factors: … (technology) to be culturally acceptable” (88)  “7 questions guide the definition of what is essential or not, including consideration of access (…) equity” (SR 31) |
| Findings | Topic/ theme (effect size) | Code | Definition | Effect size | Example quotes or fragments |

*Effect size calculated as = Frequency of topic or theme citation divided by total number of documents included in meta-synthesis
